# Impact of Preprocessing and Harmonization Methods on the Removal of Scanner Effects in Brain MRI Radiomic Features

**DOI:** 10.3390/cancers13123000

**Published:** 2021-06-15

**Authors:** Yingping Li, Samy Ammari, Corinne Balleyguier, Nathalie Lassau, Emilie Chouzenoux

**Affiliations:** 1 Laboratoire d’Imagerie Biomédicale Multimodale Paris Saclay, UMR1281 Inserm, Commissariat à l’Énergie Atomique et aux Énergies Alternatives (CEA), Centre National de la Recherche Scientifique, Université Paris-Saclay, 94805 Villejuif, France; Samy.AMMARI@gustaveroussy.fr (S.A.); Corinne.BALLEYGUIER@gustaveroussy.fr (C.B.); Nathalie.LASSAU@gustaveroussy.fr (N.L.); 2 Centre de Vision Numérique, Institut National de Recherche en Informatique et en Automatique (INRIA), Université Paris-Saclay, 91190 Gif-sur-Yvette, France; emilie.chouzenoux@centralesupelec.fr; 3 Département d’Imagerie Médicale, Gustave Roussy Cancer Campus Grand Paris, Université Paris-Saclay, 94805 Villejuif, France

**Keywords:** brain MRI radiomics, harmonization methods, intensity normalization, ComBat, reproducibility, scanner effects

## Abstract

**Simple Summary:**

As a rapid-development research field, radiomics-based analysis has been applied to many clinical problems. However, the reproducibility of the radiomics studies remain challenging especially when data suffers from scanner effects, a kind of non-biological variations introduced by different image acquiring settings. This study aims to investigate how the image preprocessing methods (N4 bias field correction and image resampling) and the harmonization methods (intensity normalization methods working on images and ComBat method working on radiomic features) help to remove the scanner effects and improve the radiomics reproducibility in brain MRI radiomics.

**Abstract:**

In brain MRI radiomics studies, the non-biological variations introduced by different image acquisition settings, namely scanner effects, affect the reliability and reproducibility of the radiomics results. This paper assesses how the preprocessing methods (including N4 bias field correction and image resampling) and the harmonization methods (either the six intensity normalization methods working on brain MRI images or the ComBat method working on radiomic features) help to remove the scanner effects and improve the radiomic feature reproducibility in brain MRI radiomics. The analyses were based on in vitro datasets (homogeneous and heterogeneous phantom data) and in vivo datasets (brain MRI images collected from healthy volunteers and clinical patients with brain tumors). The results show that the ComBat method is essential and vital to remove scanner effects in brain MRI radiomic studies. Moreover, the intensity normalization methods, while not able to remove scanner effects at the radiomic feature level, still yield more comparable MRI images and improve the robustness of the harmonized features to the choice among ComBat implementations.

## 1. Introduction

The term “radiomics” refers to the use of quantitative image features extracted from clinical images, or other clinical data, to help the diagnosis, prognosis, and the prediction of the response to treatments [1,2,3]. Instead of solely interpreting medical images visually, it identifies information invisible to the naked eyes and discovers the underlying pathological information in images through predefined image features [2,3]. As a rapid-development research field, radiomics-based data analysis has been applied to many clinical problems, such as cancer detection and classification in oncology [4,5].

However, the reproducibility and the generalization capacity of the radiomics studies remain challenging goals to achieve [6,7]. One important reason is the non-biological variations introduced by different medical centers, scanner manufacturers, scanners, protocols and so on. These unwanted non-biological variations associated with different scanning equipment and/or parameter configurations are often gathered under the term “scanner effect” [8]. Scanner effects can hinder the detection of biological and pathological information, leading to bias or unreliable conclusions. Their impact cannot be ignored in radiomic studies, especially when data are pooled from different sources.

The scanner effects also hamper the reproducibility and the multicenter studies of Magnetic Resonance Imaging (MRI) radiomics [9,10,11,12]. MRI is widely used for staging and follow-up of cancer patients, as a non-invasive and non-irradiating imaging modality. It offers extremely clear, detailed images of soft-tissue structures that other medical imaging modalities cannot achieve. However, it is very sensitive to the scanner effects introduced by different MRI equipments (magnetic field strength, gradient strength), image acquisition protocols [13,14], sequences, pixel size [15] and the signal-to-noise ratio (SNR) [16]. Besides, unlike other imaging modalities (e.g., Hounsfield Unit (HU) in Computed Tomography (CT), Standardized Uptake Value (SUV) in Positron Emission Tomography (PET)), the intensities of pixels in an MRI image do not have a clear physical meaning and lack of a standard and quantifiable interpretation [17,18,19], which makes the comparison between MRI images difficult and the reproducibility of MRI radiomics even more challenging.

Harmonization methods [20] have been proposed to remove those undesirable scanner effects or solve the incomparability among MRI images, thus improving the reproducibility of multicenter radiomic studies on MRI datasets. We can classify the harmonization methods for MRI radiomic studies into two classes, namely (i) harmonizing the MRI images before the feature extraction and (ii) harmonizing the extracted radiomic features. For the harmonization methods acting on the MRI images, we can list, among the mostly used approaches [17,18,21,22], the intensity normalization methods like Z-Score and Nyúl intensity normalization [17]. We also list some intensity normalization methods dedicated to brain MRI images like the WhiteStripe normalization [18], FCM-based normalization [21], GMM-based normalization [21], and KDE-based normalization [21], where the White Matter (WM) intensities are used as references to help the normalization. All these methods help to overcome the difficulties of MRI in interpreting the intensities between subjects, so that similar intensities will have similar tissue meaning for the standardized images. For harmonization methods working on radiomic features [23,24,25,26,27,28,29,30], a representative and widely used method is ComBat. ComBat [23] was originally proposed for the microarray expression data to remove batch effects, a type of non-biological experimental variation similar to scanner effects but observed across multiple batches in microarray experiments. In these recent years, ComBat has been transferred to many medical scenarios, showing its effectiveness to help remove scanner effects and benefit the downstream analysis in multi-site diffusion tensor imaging data [31], in cortical thickness measurements in brain MRI [8], in radiomics studies in PET [32], in CT radiomic studies [33,34], in breast MRI radiomic studies [35], in classification between cholesteatoma and middle ear inflammation in HRCT radiomics studies [36], in brain MRI radiomics studies with grade III and IV glial tumors [37], to name only a few.

Noticeably, how the ComBat harmonization influences the reproducibility of brain MRI radiomics in multicenter studies remains scarcely studied, especially how it works together with intensity normalization methods dedicated to brain MRI images. However, the multicenter brain MRI radiomic studies are very important and have significant clinical application scenarios, for example, they could help the radiologists to interpret and characterize brain tumors (such as tumor grades and pathological subtypes), to evaluate the therapeutic efficacy, and so on. Our study aims at a better understanding of the role of harmonization methods (either the intensity normalization methods working on brain MRI images, or the ComBat working on radiomic features) in the context of brain MRI radiomics, through an exhaustive study on in vitro and in vivo acquisitions. Besides, the influence of some brain MRI preprocessing methods have also been considered. Previous papers [38,39,40,41,42,43] have investigated the impact of various image preprocessing methods on brain MRI radiomic feature reproducibility. In our study, we only consider the impact of N4 bias field correction and image resampling (resampling image pixels to 1 mm × 1 mm × 1 mm) which were used as standardization in paper [19].

We evaluate the changes in magnetic field strength (1.5 Tesla (1.5T) vs. 3 Tesla (3T)) and image resolution (field of view (FOV), matrix) as the scanner effects. Let us emphasize that magnetic field strength in MRI studies is a rather important parameter, as compared to 1.5T MRI, 3T magnetic field MRI leads to increased SNR, increased image resolutions, changed T1 and T2 relaxation time constants, and increased magnetic susceptibility artifacts. Different kinds of MRI datasets are studied to make the achieved conclusions robust and trustworthy. In vitro textural phantoms have been developed to mimic homogeneous and heterogeneous tissues similar to human brain tissues with identical MRI relaxation properties. In addition, in vivo brain MRI images of healthy volunteers, as well as clinical patients from the Gustave Roussy Cancer Campus, are studied.

## 2. Materials and Methods

**Figure 1:**
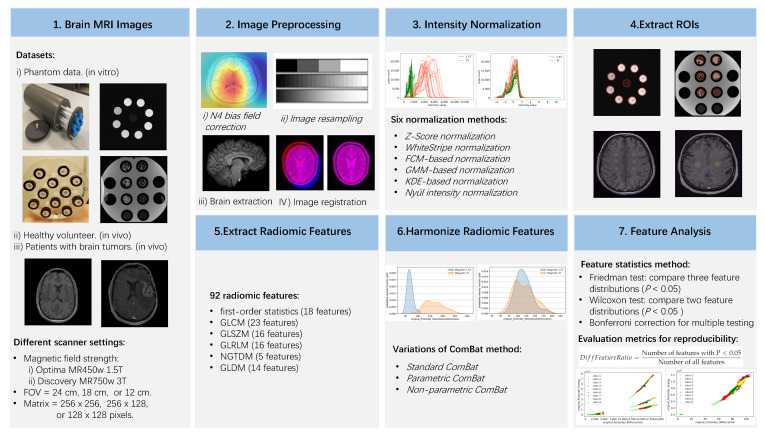
Workflow of our study. The methods in italic are the key factors we investigate for their impacts on the removal of the scanner effects. Phantom data are special, thus N4 bias field correction, brain extraction, image registration, and WM-related intensity normalization methods (WhiteStripe, FCM-based, GMM-based, and KDE-based normalization) were not used.

We firstly list the workflow of our study in Figure 1 to help us understand the materials and methods in this section.

### 2.1. MRI Images

Before introducing the MRI images used in our study, we must point out that all the image acquisition matrices mentioned later refer to the original image acquisition matrix when acquiring the MRI images. After that, all the MRI images have been resized and saved in 512×512 pixels by the MRI acquisition devices automatically. Besides, in the subsequent descriptions, we ignore the slight differences of original image matrices 300×300, 256×256, and 300×288 pixels present in our data, and just describe them as 256×256 pixels for simplicity.

#### 2.1.1. Phantoms

A homogeneous phantom was designed to mimic cerebrospinal fluid and opacified blood vessels, while a heterogeneous phantom was designed to mimic the brain white matter. Both the homogeneous and heterogeneous phantoms were scanned by two clinical MRI scans (Optima MR450w 1.5T and Discovery MR750w 3T, both from GE Healthcare, Milwaukee, WI, USA), with different FOVs (24, 18, and 12 cm) and different matrices (256×256, 256×128, and 128×128 pixels). Only T1 clinical sequences were considered. More details about the image acquisition devices and protocols can be found in paper [12] by Ammari et al. who studied the same phantom dataset.

#### 2.1.2. Healthy Volunteers

T1 clinical brain MRI sequences were collected from 6 healthy volunteers to investigate the impact of different magnetic field strengths (1.5T vs. 3T) on the reproducibility of brain MRI radiomics. Each volunteer has two MRI images, scanned by two different MRI devices (Optima MR450w 1.5T and Discovery MR750w 3T) with the fixed FOV of 24 cm and image matrix of 256×256 pixels, within a 40 min time interval. Meanwhile, another volunteer was scanned by Optima MR450w 1.5T scanner for T1 clinical sequences, with the fixed matrix of 256×256 pixels but two different FOVs (24 cm vs. 18 cm) to study the impact of FOV, and with the fixed FOV of 24 cm but three different matrices (256×256 vs. 256×128 vs. 128×128 pixels), with the aim to investigate the influence of matrix. One can refer to paper [12] by Ammari et al. for more details about the MRI image acquisition devices and protocols.

#### 2.1.3. Clinical Patients

Twenty patients with brain tumors treated at the Gustave Roussy Cancer Campus were also collected to study the magnetic field strength in brain MRI radiomics. For each patient, we selected the T1 sequences of two MRI scans with the shortest time interval among all the available clinical MRI scans, one scanned by Optima MR450w 1.5T and the other scanned by Discovery MR750w 3T, with the same FOV (24 cm) and the same image matrix (256×256 pixels). The average and maximum time interval between the two scans of the same patient are 147 days and 376 days, respectively, with statistics on all the 20 patients.

### 2.2. Region of Interests (ROIs)

For MRI images of the homogeneous phantom, we selected 70 continuous slices (51th to 120th slice) where all the nine homogeneous tubes clearly appear, in order to extract ROIs. For each slice, nine circular 2D ROIs corresponding to the nine homogeneous tubes were extracted, using a fixed radius size (14 pixels for FOV = 24 cm, 18 pixels for FOV = 18 cm, and 28 pixels for FOV = 12 cm). For MRI images of heterogeneous phantom, 30 continuous slices (21th to 50th) were selected. Since only six heterogeneous tubes were visible for FOV = 12 cm, we only extracted six circular 2D ROIs per slice with fixed radius size (22 pixels for FOV = 24 cm, 28 pixels for FOV = 18 cm, and 42 pixels for FOV = 12 cm).

For the brain MRI images of the healthy volunteers, we manually extracted 60 circular 2D ROIs per T1 image sequence by 3D slicer (version 4.11.20200930), namely, 20 white matter (WM) ROIs and 20 gray matter (GM) ROIs in the transverse plane, and 20 corpus callosum (CC) ROIs in the sagittal plane. When investigating the impact of magnetic field strength, the ROIs were manually extracted on the 1.5T MRI images and then copied to the corresponding 3T images, which have been registered to the corresponding 1.5T images using rigid transformation [44,45,46] by ANTsPy (https://antspy.readthedocs.io/en/latest/, accessed on 30 November 2020, version 0.1.7). Similarly, when studying the impact of FOV and matrix, the brain MR images of the same volunteer acquired with different FOVs and matrices were also registered using rigid transformation by ANTsPy firstly. After that, the ROIs manually acquired on the fixed images were copied to the registered images. In this way, we can ensure that the extracted ROIs come from the same brain regions of the same volunteers, thus the differences among them can only be due to different image acquisition settings such as different magnetic field strengths, FOVs and matrices.

Similarly, for each patient, the MRI images from the same patient were firstly registered using rigid registration by ANTsPy. Then, circular 2D ROIs were extracted on the fixed images using 3D slicer and copied to the registered images. The fixed images used here for registration were the T1 Gado sequences of the 1.5T brain MRI images (not studied in our paper), the T1 sequences of 1.5T and 3T MRI images studied in our paper were registered to the corresponding fixed images. Besides, it is difficult to tell whether the pathological information in the tumor regions have changed or not across the MRI images of the same patient by the naked eyes, thus we only extract ROIs from healthy WM regions, with the basic hypothesis that the healthy WM regions had no substantive hidden changes on radiomic features if not observed visually in images. Finally, six circular 2D ROIs were extracted from healthy WM regions for each brain MRI image.

Since the impact of the image resampling preprocessing was studied, we must emphasize here that all the ROIs for the resampled images were automatically acquired by resampling the original ROIs to 1 mm × 1 mm × 1 mm using ANTsPy with the nearest neighbor interpolation strategy.

### 2.3. Radiomic Features

Using the above processing, all the ROIs are two-dimensional. For each 2D ROI, 92 radiomic features [47] were extracted from the original MRI images without any image filters applied, using pyradiomics [48] (https://pyradiomics.readthedocs.io/en/latest/, accessed on 30 November 2020, version 3.0.1), an open-source Python package for the extraction of radiomic features from medical imaging. These radiomic features consist of 18 first-order statistics features, 23 Gray Level Cooccurence Matrix (GLCM) features, 16 Gray Level Run Length Matrix (GLRLM) features, 16 Gray Level Size Zone Matrix (GLSZM) features, 5 Neighbouring Gray Tone Difference Matrix (NGTDM) features and 14 Gray Level Dependence Matrix (GLDM) features. When extracting radiomic features, we directly used the fixed bin number strategy and took 32 bins for discretization, motivated by its good performance in [19].

### 2.4. Image Preprocessings

The influences of the two image preprocessing methods are considered. The first one is N4 bias field correction [49], one of the most popular methods to correct low-frequency intensity non-uniformity (also known as bias, inhomogeneity, illumination nonuniformity, or gain field) present in the MRI images. N4 bias field correction was implemented by ANTsPy, with the shrink factor for multi-resolution correction set to 2, the maximum number of iterations set to 100, and other parameters using default values. Note that we did not apply N4 bias field correction for phantom data, because the non-uniformity among experiment tubes are normal and should not be corrected. The second preprocessing method considered is image resampling, which was implemented by ANTsPy using b-spline interpolation strategy, ensuring that all the MRI images have the same voxel size, namely 1 mm × 1 mm × 1 mm in our study.

### 2.5. Harmonization Methods

The most important aim of this study is to investigate how the harmonization methods (either working on the brain MRI images or on the radiomic features) would influence the removal of scanner effects and improve the reproducibility of brain MRI radiomics. For this purpose, six image intensity normalization methods are studied for brain MRI images. Let I(x) and $I_{norm}\left(x\right)$ be the intensities of the raw MR image and the normalized MR image, respectively, then we can group the intensity normalization methods into three classes as below, according to their definitions [21].

(1) Z-Score and WhiteStripe normalization [18]:
  (1) $$I_{norm}\left(x\right)=\frac{I(x)-\mu }{\sigma }$$
  where μ and σ correspond to mean and the standard deviation of the intensities inside the brain mask for Z-Score and inside a defined “White Stripe” region for WhiteStripe normalization.
(2) FCM-based, GMM-based, and KDE-based normalization [21]:
  (2) $$I_{norm}\left(x\right)=\frac{c\cdot I(x)}{\mu },$$
  where μ corresponds to the WM mean estimated based on fuzzy C-Means (FCM) or Gaussian Mixture Model (GMM), and *c* is a constant that determines the WM mean after normalization for FCM-based and GMM-based normalization. For KDE-based normalization, μ is the estimated WM peak by Kernel Density Estimate (KDE), and *c* is a constant determining the WM peak after normalization.
(3) Nyúl normalization [17,50]: also called piecewise linear histogram matching normalization, learns a standard image histogram from a set of images, and then linearly maps the intensities of each image to this standard image histogram.

Among these six intensity normalization methods, only Z-Score and Nyúl normalization are possible for the phantom data since the others are based on the WM intensities, thus dedicated to brain MRI images. All these intensity normalization methods can be regarded as harmonization methods acting on MRI images, and their detailed definitions can be found in [21]. Note that brain masks were needed when performing the intensity normalization. We directly used the public Python codes for brain mask extraction (https://pypi.org/project/deepbrain/, accessed on 30 November 2020, version 0.1) and for intensity normalization (https://github.com/jcreinhold/intensity-normalization, accessed on 2 December 2020, version 1.4.3). For phantom data, the ROI masks were passed as normalization masks for Z-Score.

Regarding the ComBat method which works on the extracted radiomic features, three variants would be investigated, standard ComBat without using Empirical Bayes (EB), parametric ComBat using parametric EB and non-parametric ComBat using non-parametric EB for more robust data adjustments. The public Python codes (https://github.com/Jfortin1/neuroCombat, accessed on 30 November 2020, version 0.2.7) were used in our experiments. Note that we do not consider the covariates of interest (i.e., X=0) in ComBat method, because all of our data are pair-wised, namely, they are from the same individuals or same phantoms, but with different acquisition settings.

### 2.6. Evaluation Metrics for the Radiomics Reproducibility

We have isolated the same ROIs for each MRI image pair which means the images from the same phantom or the same person, scanned using different magnetic field strengths, FOVs or matrices. If the scanner effects or non-biological variations do not exist, then the distributions of the features extracted from the same ROIs should be highly similar. To measure this similarity, a Friedman test [51] was used for the comparison between three feature distributions (for example, features from matrix 256×256, 256×128 and 128×128 pixels), whereas the Wilcoxon test [52] was used for the comparison between two distributions (for example, features from 1.5T and 3T images). Bonferroni correction (https://www.statsmodels.org/dev/generated/statsmodels.stats.multitest.multipletests.html, accessed on 30 November 2020, version 0.12.1) [53,54,55] was used for multiple testing corrections. A *P* value of less than 0.05 indicates the significantly different feature distributions, while a *P* value greater than 0.05 in our study means that the scanner effects do not exist. To have an overall evaluation of the ability of the harmonization methods to remove scanner effects in the radiomic feature level, we introduce the ratio of the radiomic features with *P*<0.05 to the overall radiomic features as the evaluation metric, with the name DiffFeatureRatio for easy reference in our paper, as described in Formula (3). When calculating the DiffFeatureRatio, all the 92 features for each ROI for each MRI image are counted. The values of DiffFeatureRatio lie in [0,1], where 0 and 1 correspond to the perfect and worst harmonization efficiency, respectively.

(3) $$DiffFeatureRatio=\frac{\mathrm{Number}\;\mathrm{of}\;\mathrm{features}\;\mathrm{with}\;P<0.05}{\mathrm{Number}\;\mathrm{of}\;\mathrm{all}\;\mathrm{features}}$$

**Figure 2:**
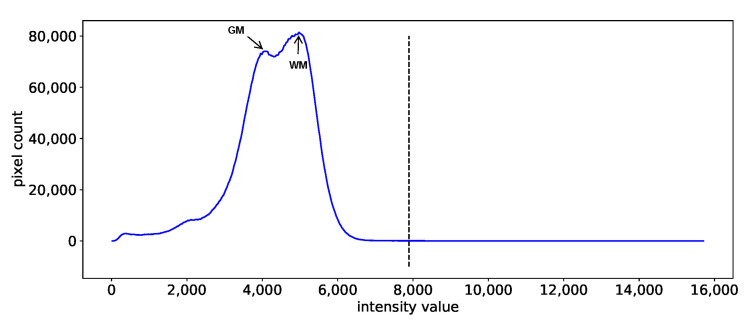
Example of an image histogram of the T1-weighted brain MRI image. Here GM and WM represent the gray matter and white matter of the brain, respectively.

In addition to the quantitative evaluation metrics DiffFeatureRatio, some figures are also used to help the analysis. We visualize the first three principal components of Principal Component Analysis (PCA) [56,57] to help verify whether the useful biological information is preserved after different preprocessing and harmonization methods. Note that, before performing PCA, we standardize the radiomics features to ensure a zero mean and unit variance for each feature. The histograms of the brain MRI images are also used to help the analysis. As illustrated in Figure 2, the image histogram (in blue color) of a healthy brain MRI image within the brain mask region exhibits some intensity peaks. For T1-weighted brain MRI images, the peaks associated with the greatest intensity and the second greatest intensity correspond to WM and GM, respectively. In our study, we use 512 bins for the brain MRI histogram discretization. For better illustration, we only show part of the histogram (for example, the area to the left of the black dashed line) which carries most of the histogram information. We refer to it simply as a brain MRI image histogram in the subsequent descriptions.

## 3. Results

**Figure 3:**
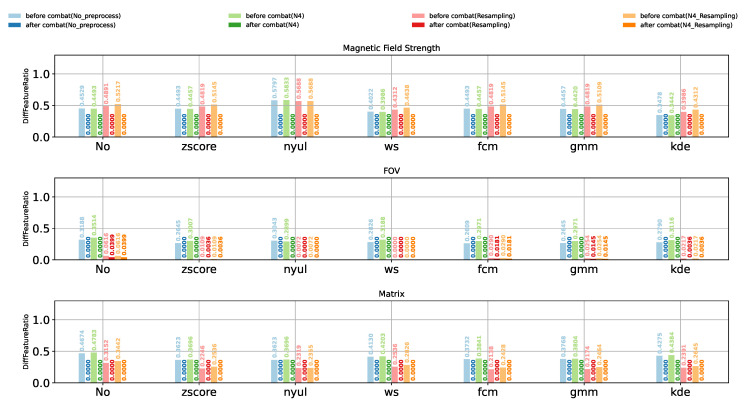
Impact of different preprocessing and harmonization methods for healthy volunteer data.

**Figure 4:**
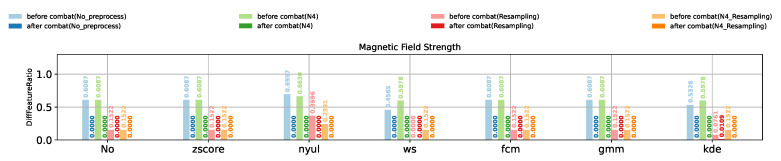
Impact of different preprocessing and harmonization methods for clinical patient data.

**Figure 5:**
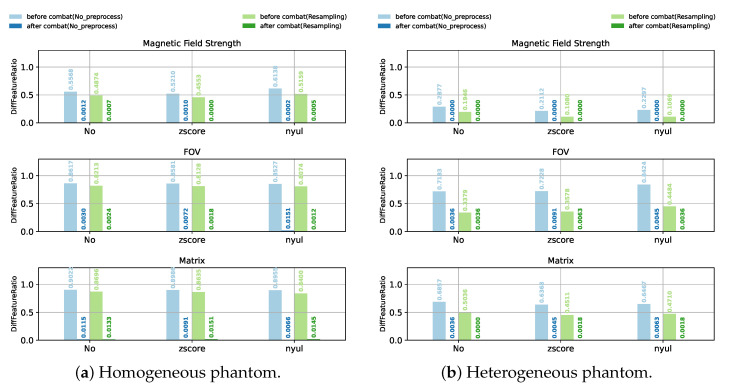
Impact of different preprocessing and harmonization methods for (**a**) homogeneous phantom data and (**b**) heterogeneous phantom data. N4 bias field correction was not considered for phantom data.

In order to compare the impact of different combinations of preprocessing and harmonization methods, a large set of experiments were conducted. For the sake of better illustrative and intuitive comparisons among the experiment results we obtained, the values of DiffFeatureRatio representing the ratio of features who have significantly different feature distributions among different scanner settings are shown using bar plots. Figure 3, Figure 4 and Figure 5 show the DiffFeatureRatio results of the healthy volunteer data, the clinical patient data and the phantom data, respectively, considering different kinds of scanner effects either introduced by different magnetic field strengths, different FOVs or different matrices. In these figures, we display the different intensity normalization methods on the horizontal axis, “No” meaning without any intensity normalization, and the values above the bar plots represent the corresponding DiffFeatureRatio. Here, the parametric ComBat method was applied for each tissue class (WM, GM, CC, etc.), separately. Based on these three figures, we can now discuss in detail how the image preprocessing methods and the harmonization methods influence the removal of the scanner effects thus improving the radiomic feature reproducibility.

### 3.1. Impact of N4 Bias Field Correction on the Removal of Scanner Effects

**Figure 6:**
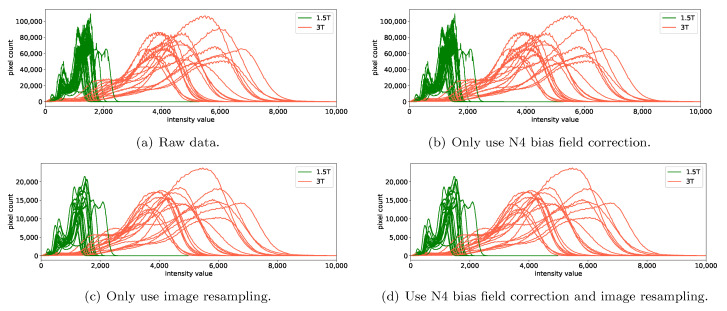
Impact of preprocessing methods on image histograms of brain MRI images of patient data, scanned with 1.5T and 3T. The subfiures show the image histograms of the MRI images (**a**) without using any preprocessing methods (raw data), (**b**) with only using the N4 bias field correction, (**c**) with only using the image resampling (1 mm × 1 mm × 1 mm), and (**d**) with using both the N4 bias field correction and image resampling. Note that the intensity normalization methods and ComBat method were not used here.

N4 bias field correction corrects the low-frequency intensity non-uniformity present in the MRI images. We did not consider the N4 bias field correction for phantom data. By inspecting the results of healthy volunteer data in Figure 3 and the patient data in Figure 4, one can see that N4 bias field correction has no obvious impact on the DiffFeatureRatio values in most cases, regardless of whether image resampling, intensity normalization, or ComBat were applied or not. This phenomenon can be expected, because MRI images of the healthy volunteers and the patient data in our study did not suffer from obvious bias field effects, thus the bias-corrected images were similar to the original images. Figure 6 shows how the N4 bias field correction and image resampling influence the image histograms of the MRI images of patient data, with scanner effects caused by different magnetic field strengths (1.5T vs. 3T). As expected, the image histograms are almost the same after applying N4 bias field correction. We though infer that the results would be different for MRI images affected with severe bias field effects, and we leave it to be further investigated in future studies. In the following experiments, we do not use N4 bias field correction as a preprocessing method as it has no obvious impact on our dataset.

### 3.2. Impact of Image Resampling on the Removal of Scanner Effects

Image resampling ensures the same voxel size (1 mm × 1 mm × 1 mm in our study) in MRI images. From the DiffFeatureRatio values presented in Figure 3, Figure 4 and Figure 5, we observe that image resampling has a clearly different impact on the radiomic feature reproducibility for different dataset and different scanner effects. For the healthy volunteer data with different magnetic field strengths, image resampling leads to slightly bigger DiffFeatureRatio values, meaning more significantly different feature distributions. For other cases, namely for patient data and phantom data, as well as healthy volunteer data with different FOVs and matrices, image resampling helps to decrease DiffFeatureRatio values. Especially for healthy volunteer data with different FOVs, image resampling helps to reduce the DiffFeatureRatio from 0.3188 to 0.0616 for the raw data, meaning that most of the scanner effects introduced by different FOVs have been removed.

Since image resampling works on MRI images, we also display the image histograms. We found that the image histograms of the MRI images may not be smooth and may exhibit oscillations, as shown in Figure 6a. Image resampling may help to alleviate the oscillations, as for our healthy volunteer data and patient data, with an example shown in Figure 6c. Meanwhile, the shapes of the image histograms and thus the biological information in MRI image level are well kept after applying image resampling. However, image resampling could also possibly aggravates the oscillation phenomenon present in image histograms, as for our homogeneous phantom and heterogeneous phantom data. We guess that the alleviation or aggravation of the oscillation phenomenon in image histograms corresponds to the alleviated or aggravated noise caused by the interpolation during image resampling. The increase or decrease of the DiffFeatureRatio values may not relate to the oscillations in image histograms, but relate to whether the image histograms are more similar after image resampling.

Noticeably, image resampling could not shift the mean intensity values, thus could not remove such scanner effects in MRI image level, resulting in still a large gap between the mean intensity values of 1.5T and 3T after image resampling (see Figure 6c).

### 3.3. Impact of Intensity Normalization Methods on the Removal of Scanner Effects

**Figure 7:**
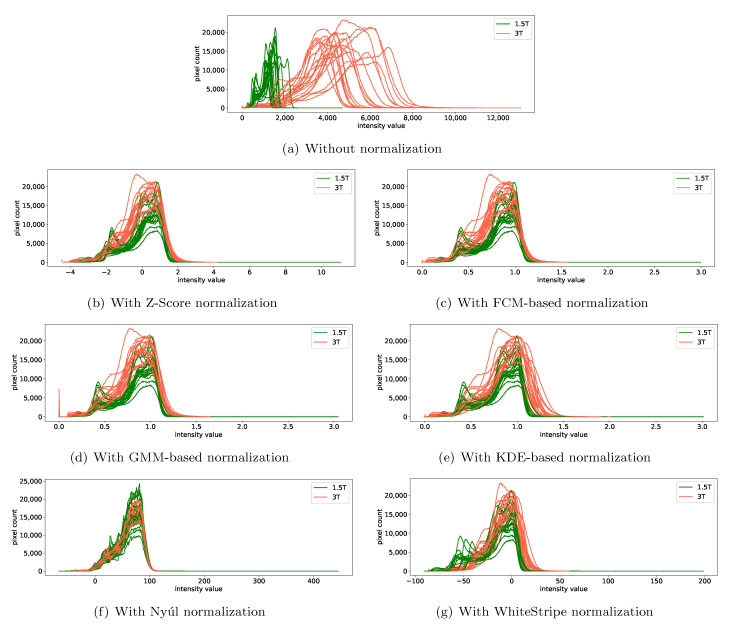
Impact of the intensity normalization methods on the image histograms of the brain MRI images of the clinical patient data, scanned by 1.5T and 3T scanner. The subfigures show the image histograms of the MRI images (**a**) without using any intensity normalization methods, (**b**) with using the Z-Score normalization, (**c**) with using the FCM-based normalization, (**d**) with using the GMM-based normalization, (**e**) with using the KDE-based normalization, (**f**) with using the Nyúl normalization and (**g**) with using the WhiteStripe normalization. Note that here image resampling were used as preprocessing method to alleviate the slight oscillation in original image histograms.

Intensity normalization methods, acting on MRI images, work as types of harmonization methods at the MRI image level. According to the DiffFeatureRatio values shown in Figure 3 for healthy volunteer data, Figure 4 for patient data, and Figure 5 for phantom data, we observe that the impact of the intensity normalization methods on the radiomic feature reproducibility is not obvious compared to not using any intensity normalization methods. Similarly, we plot the image histograms of the normalized images to help the analysis. The image histograms of the normalized images by different kinds of normalization methods for the patient data are shown in Figure 7. Obviously, the image histograms of the normalized images by Nyúl normalization seem to be non-smooth and noisy as shown in Figure 7f. Similar noise was also observed in the image histograms of the Nyúl normalized images for healthy volunteer data with different magnetic field strengths, which may be the reason for the bigger DiffFeatureRatio values compared to the other normalization methods in Figure 3. The non-smooth and noisy phenomenon present in the image histograms of Nyúl normalized images might be due to the fact that the intensity histograms differ significantly for MRI images acquired by different scanners (with 1.5T and 3T magnetic field strengths), or for patients with different tumors (different tumor numbers, tumor textures or tumor sizes), thus making it difficult for the standard histogram learning and histogram matching in the process of Nyúl normalization. The other five intensity normalization methods involved in our study could help to eliminate the big gap between the mean intensity values of 1.5T and 3T images, ending with similar shapes and peak values. It indicates that the main information that existed in the MRI images are kept, and the intensity values of the MRI images are comparable now. Surprisingly, the scanner effects at the radiomic feature level still exist after applying these intensity normalization methods, shown as the still big DiffFeatureRatio values after intensity normalization in Figure 3, Figure 4 and Figure 5.

### 3.4. Impact of ComBat Method on the Removal of Scanner Effects

From the above analysis, the intensity normalization methods could only make the MRI images comparable among different scanner settings, the radiomic features still have a very poor reproducibility after intensity normalization. Now we focus on the impact of the ComBat method, a harmonization method working on radiomic features. Obviously, for all the healthy volunteer data (Figure 3), the patient data (Figure 4) and the phantom data (Figure 5), no matter the scanner effects introduced by different magnetic field strengths, FOVs or matrices, no matter whether N4 bias field correction, image resampling or intensity normalization were applied, as long as parametric ComBat was applied, the DiffFeatureRatio values always tend to zero, meaning that most radiomic features cannot be detected to have significantly different feature distributions among different scanner settings. It indicates that ComBat is the essential and key factor in radiomic feature level to remove the scanner effects and improve the radiomic feature reproducibility.

### 3.5. Impact of the Preprocessing and Harmonization Methods on Keeping the Useful Biological Information

**Figure 8:**
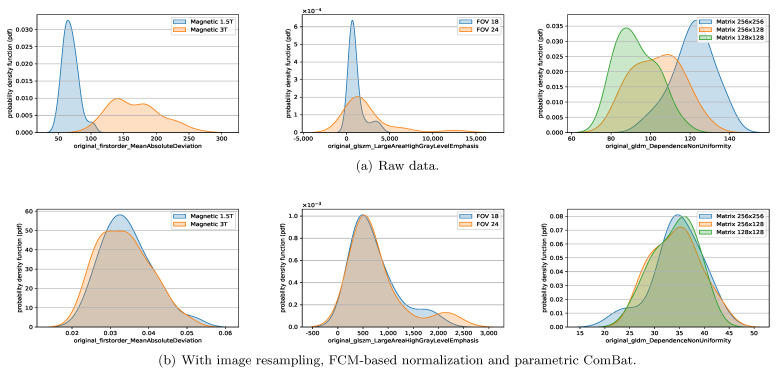
Histograms of example radiomic features of the raw data and the harmonized data of healthy volunteers, with different magnetic field strengths (first column), FOVs (second column) and matrices (third column). (**a**) The histograms of the example features of the raw data. (**b**) The histograms of the example features after applying the image resampling, FCM-based normalization and parametric ComBat methods.

The above analyses and conclusions are based on the values of DiffFeatureRatio, a metric we define based on the Friedman test and the Wilcoxon test. In essence, it can only give insight into whether the feature distributions are significantly different or not. If the feature distributions cannot be detected significantly different among different scanner settings, we can assume the scanner effects, namely, the non-biological information introduced by different MRI image acquisition settings, have been successfully removed. Figure 8 shows some example features of the healthy volunteer data. Apparently, after applying the preprocessing and harmonization methods (mainly due to the ComBat), the histograms of the radiomic features extracted from the same ROIs but acquired by different scanner settings overlap more compared to the raw data, which demonstrates that the scanner effects have been successfully removed.

**Figure 9:**
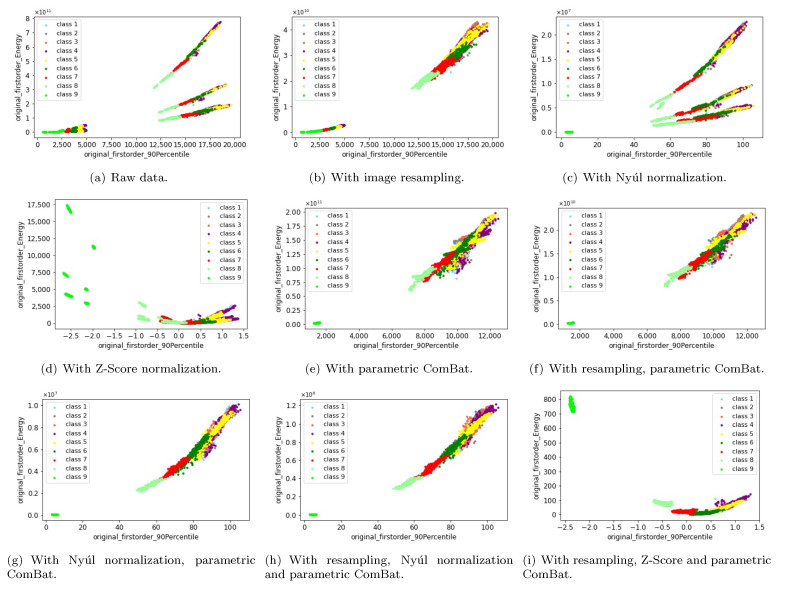
Visualize two features (“90th percentile” and “energy”) of the homogeneous phantom in a 2D plane, with different preprocessing and harmonization methods. Here “class 1” to “class 9” correspond to the nine homogeneous phantom tubes. The subfigures show the two example features acquired from MRI images (**a**) without applying any preprocessing and harmonization methods (raw data), (**b**) with only applying the image resampling, (**c**) with only applying the Nyúl normalization, (**d**) with only applying the Z-Score normalization, (**e**) with only applying the parametric ComBat, (**f**) with applying the resampling and parametric ComBat, (**g**) with applying the Nyúl normalization and parametric ComBat, (**h**) with applying the resampling, Nyúl normalization and parametric ComBat, and (**i**) with applying the resampling, Z-Score and parametric ComBat.

**Figure 10:**
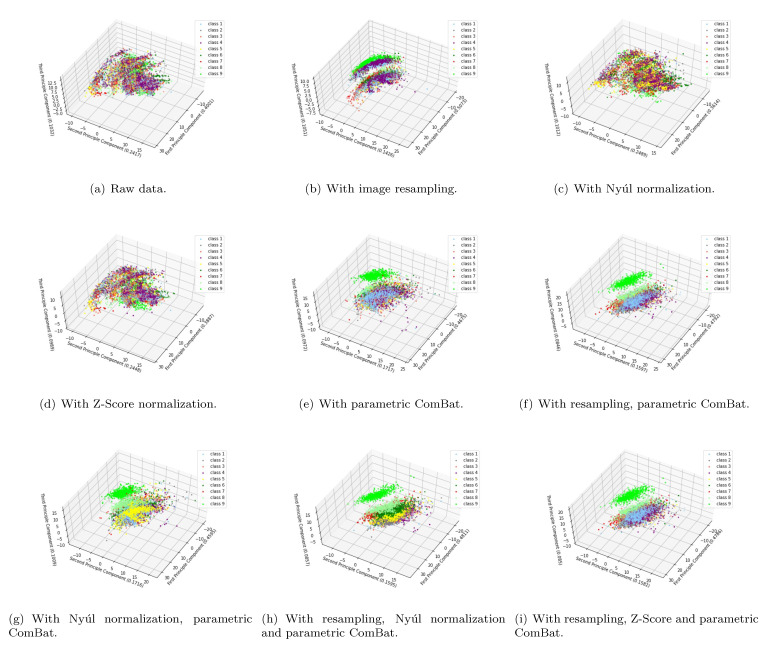
Visualize the first three principal components of PCA of the homogeneous phantom data in a 3D plane, with different preprocessing and harmonization methods. Here “class 1” to “class 9” correspond to the nine homogeneous phantom tubes. The subfigures show the two example features acquired from MRI images (**a**) without applying any preprocessing and harmonization methods (raw data), (**b**) with only applying the image resampling, (**c**) with only applying the Nyúl normalization, (**d**) with only applying the Z-Score normalization, (**e**) with only applying the parametric ComBat, (**f**) with applying the resampling and parametric ComBat, (**g**) with applying the Nyúl normalization and parametric ComBat, (**h**) with applying the resampling, Nyúl normalization and parametric ComBat, and (**i**) with applying the resampling, Z-Score and parametric ComBat. Note that the identifiability between different classes is better visible in the animation form, see https://github.com/Yingping-LI/MRI_Radiomics/blob/main/preprocessing_and_harmonization.md, accessed on 11 June 2021.

Another important question one may ask is whether the useful biological information existed in the original MRI images are successfully kept after applying these preprocessing and harmonization methods. If not, then the harmonized features would be meaningless. To answer this question, we use the homogeneous phantom data as an example, and visualize two example features (Figure 9) and the first three principal components of PCA (Figure 10) in a 2D/3D plane, to discuss how these preprocessing and harmonization methods influence the biological information. In these two figures, the 9 colors correspond to nine different pattern classes (nine homogeneous phantom tubes), and the subclasses of each color correspond to the 10 different scanner settings (involving different magnetic field strengths, FOVs and matrices). For each color (pattern class), if all the subclasses merge into one, then it indicates that the non-biological information have been successfully removed. If each color (each pattern class) is identifiable from the others in the raw data, then we can only say the useful biological information are well kept if they are still identifiable after applying the preprocessing or harmonization methods. Only when the non-biological information are successfully removed while at the same time the biological information are well-kept, we can finally conclude that the harmonization results are satisfying and the scanner effects have been perfectly removed. From the two feature examples visualized in Figure 9 and the first three principal components of PCA in Figure 10, we can obtain some interesting results. Firstly, image resampling helps to decrease the number of the subclasses for each color, but there still exists some subclasses for each color (possibly corresponding to 1.5T and 3T magnetic field strengths), as shown in Figure 9b and Figure 10b. It indicates that image resampling would help to remove part of the scanner effects. Secondly, when only applying Nyúl or Z-Score normalization, there are still many subclasses for each color (Figure 9c,d and Figure 10c,d), meaning the scanner effects still exist. Thirdly, parametric ComBat, or parametric ComBat combined with image resampling, helps to make the subclasses merge into one, having similar feature mean values, but the variances of the radiomic features seem to still be different (shown in Figure 9e,f). Fourthly, when applying both the Nyúl normalization and parametric ComBat, no matter if using image resampling or not, the harmonization results are satisfying, meaning the non-biological information are removed and the useful biological information are well kept, as shown in Figure 9g,h for feature examples, in Figure 10g,h for PCA results. Lastly, we found that Z-Score intensity normalization changes the relative relationships of the radiomic features (Figure 9i), but still preserves identifiability between patterns (Figure 9i and Figure 10i), which indicates that useful biological information is well-kept.

**Figure 11:**
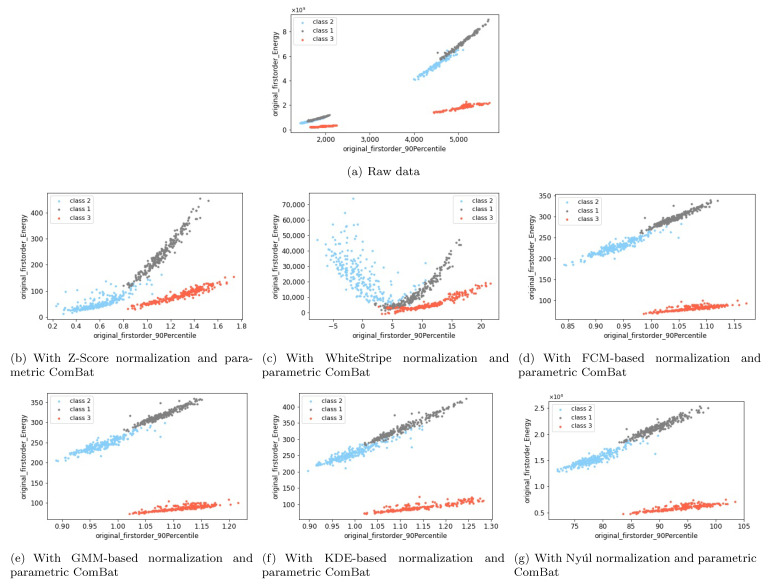
Visualize two example features in a 2D plane to compare different intensity normalization methods for the healthy volunteer data with different magnetic field strengths (1.5T vs. 3T). Here, “class 1”, “class 2” and “class 3” correspond to WM, GM and CC ROIs, respectively. The subfigures show the two example features acquired from MRI images (**a**) without applying any preprocessing and harmonization methods (raw data), (**b**) with applying the Z-Score normalization and parametric ComBat. (**c**) with applying the WhiteStripe normalization and parametric ComBat, (**d**) with applying the FCM-based normalization and parametric ComBat, (**e**) with applying the GMM-based normalization and parametric ComBat, (**f**) with applying the KDE-based normalization and parametric ComBat, and (**g**) with applying the Nyúl normalization and parametric ComBat.

We mostly use the homogeneous phantom to illustrate the results, because it was scanned with multiple scanner settings (10 scanner settings) and have multiple pattern classes (nine phantom tubes), at the same time some pattern classes are identifiable from the others. Thus more interesting phenomenons could be observed. However, only Z-Score and Nyúl normalization are possible for homogeneous phantom because the other intensity normalization methods (WhiteStripe, FCM-based, GMM-based, and KDE-based normalization) are dependent on the WM regions. We use the healthy volunteer data to have an overall evaluation of all the six intensity normalization methods, since only one tissue class (WM) was studied in patient data thus the identifiability between different pattern classes was not possible. Figure 11 shows two example features of the healthy volunteer data with 1.5T and 3T magnetic field strengths, and different intensity normalization methods were applied. Consistently with the conclusions from the homogeneous phantom, when working with ComBat method, both Z-Score and Nyúl normalizations can work well to remove scanner effects and keep the biological information (Figure 11b,g), despite that the relative relationship of the features may change. The same conclusion holds for the other four intensity normalization methods (WhiteStripe, FCM-based, GMM-based and KDE-based intensity normalization), as shown in Figure 11. In Figure 11, image resampling was not used. Actually, the conclusions regarding the intensity normalization methods were the same when image resampling was used.

**Figure 12:**
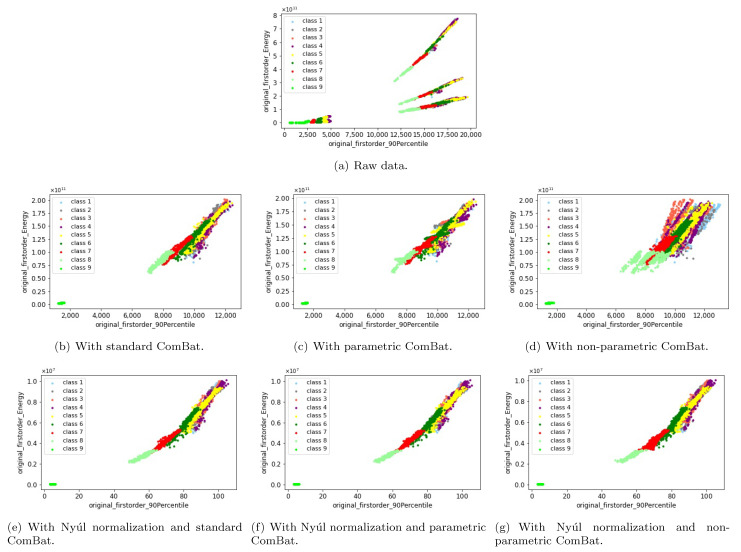
Visualize two features (“90th percentile” and “energy”) of the homogeneous phantom data in a 2D plane, to investigate different variants of ComBat method. Here “class 1” to “class 9” correspond to the nine homogeneous phantom tubes. The subfigures show the two example features acquired from MRI images (**a**) without applying any preprocessing and harmonization methods (raw data), (**b**) with applying the standard ComBat, (**c**) with applying the parametric ComBat, (**d**) with applying the non-parametric ComBat, (**e**) with applying the Nyúl normalization and standard ComBat, (**f**) with applying the Nyúl normalization and parametric ComBat, and (**g**) with applying the Nyúl normalization and non-parametric ComBat.

We also compare three different variants of ComBat method, namely, standard ComBat, parametric ComBat and non-parametric ComBat. The two example features before and after ComBat harmonization are shown in Figure 12. If there was no preprocessing and intensity normalization methods, standard ComBat works slightly better than parametric ComBat, and much better than non-parametric ComBat. As long as Nyúl normalization (or Z-Score) was used, then both standard ComBat, parametric ComBat and non-parametric ComBat can provide satisfying harmonization results, with non-biological scanner effects successfully removed and useful biological information well kept. In other words, the intensity normalization methods may make the harmonization results more robust on choosing different kinds of ComBat variants.

## 4. Discussion

In this paper, we mainly aim to investigate how the image preprocessing and harmonization methods influence the removal of scanner effects and the radiomic feature reproducibility. Two image preprocessing methods were studied, namely N4 bias field correction and image resampling (1 mm × 1 mm × 1 mm). Regarding the harmonization methods, both the harmonization methods working on MRI images (six intensity normalization methods) and the harmonization method working on radiomic features (ComBat method) were studied. The scanner effects in our study came from different MRI scanners (Optima MR450w 1.5T vs. Discovery MR750w 3T) represented by different magnetic field strengths (1.5T vs. 3T) in our paper, as well as different acquisition parameters including different FOVs and matrices. Different kinds of data were studied, including in vitro data and in vivo data, to make our conclusions robust and trustworthy. The in vitro data consists of homogeneous phantoms to mimic cerebrospinal fluid and opacified blood vessels, and the heterogeneous phantoms to mimic brain white matter. The in vivo data are composed of brain MRI images collected from some healthy volunteers, as well as some brain MRI images from clinical patients who were treated in a cancer hospital, Gustave Roussy Cancer Campus.

Regarding the image preprocessing methods, N4 bias field correction has no obvious impact on the reproducibility of radiomic features in our healthy volunteer and patient data experiments. It may be explained by the fact there is no obvious bias field effects exist in these brain MRI images. Without demonstrated it in detail in our results section, we recommend the users carefully adjust the parameters of N4 bias field correction, to prevent that some important information existed in image histograms may be lost. For example, by using N4 bias field correction by ANTsPy with default parameters, the WM and GM peaks merge into one for our healthy volunteer data and patient data. Regarding the image resampling, we observe that when resampling the image voxels to 1 mm × 1 mm × 1 mm, some of the scanner effects could be removed in some cases. By plotting the image histograms, the oscillations in the image histograms were observed to be alleviated or aggravated possibly because of the alleviated or aggravated noise caused by the interpolation during image resampling.

We investigated six intensity normalization methods for brain MRI image standardization. Based on their definitions [21], we classify them into three classes as mentioned before: (1) Z-Score and WhiteStripe; (2) FCM-based, GMM-based and KDE-based normalization; (3) Nyúl intensity normalization. All these intensity normalization methods can make the brain MRI images comparable among subjects by bringing the image intensities into a common scale, thus overcoming the weakness of the intensity of MRI images lacking standard and quantifiable interpretation. However, surprisingly, the feature reproducibility has not been significantly improved after applying any of these six intensity normalization methods, indicating that only applying intensity normalization on MRI images might not be enough in radiomics studies. Our results additionally show that the ComBat method is critical and essential to remove scanner effects and improve the feature reproducibility, at the brain MRI radiomics level. In our experiments, most of the scanner effects, introduced by either different magnetic field strengths, FOVs or matrices, were removed as long as the ComBat method was applied.

When inspecting the image histograms of the normalized images, Nyúl normalization was found to make the image histograms noisy for healthy volunteer data and patient data. The reason may be due to the tumor diversities (e.g., tumor numbers, tumors sizes, and tumor textures), or the significantly different intensity values between brain MRI images acquired with different scanner settings (for example, 1.5T vs. 3T), thus influencing the standard image histogram learning or histogram matching during Nyúl normalization. The intensity normalization methods may change the relative relationship among different pattern classes. They will still preserve useful biological information. Besides, regarding three different variants of ComBat method (standard ComBat, parametric ComBat and non-parametric ComBat), by applying the intensity normalization methods, the harmonization result appeared more robust to the choice among variants of ComBat.

Some results achieved in our paper can be found to be consistent with the previously published papers. For example, Um et al. [38] investigated the impact of image preprocessing on the scanner dependence of radiomic features in glioblastoma multiforme datasets. Among these image preprocessing methods, they reported that bias field correction did not have a significant impact on the scanner effects caused by different scanner manufactures or different scanner magnetic field strengths. We achieved the same conclusion on our datasets, but claimed that it may be due to the non-obvious bias field effects. Besides, the authors also reported that isotropic resampling (1 mm × 1 mm × 1 mm) decreased the number of features dependent on different magnetic field strengths (1.5T vs. 3T), while we observed the same phenomenon on our phantom data and patient data, despite a slightly increased amount of magnetic field strength dependent features for volunteer data. Carré et al. [19] reported in their paper that, with the fixed bin number discretization strategy, WhiteStripe and Z-Score methods achieved the same percentages of the robust features with the raw images, for both T1w-gd (post-contrast T1-weighted) and T2w-flair MRI sequences. We observed similar results on our T1-weighted sequence datasets (with 32 fixed bins for discretization). At the same time, in paper [19], Nyúl method provided the higher percentage of robust textural features for the T1w-gd sequence but a lower percentage for T2-flair sequence, compared to images without any normalization. We also observed a lower percentage of robust features for Nyúl normalization on our T1-weighted sequences of healthy volunteer data and patient data with different magnetic field strengths. Orlhac et al. [33] studied the CT phantom and patients with lung cancers, concluding that most of the non-biological differences related to CT scanners can be corrected by the ComBat method. This conclusion is quite consistent with the results in our paper, although on different medical image modalities.

The work in our paper differs from the studies in the previously published papers. On the one hand, our paper studies both the intensity normalization methods working on MRI images and the ComBat method working on radiomic features, and how they work together to remove the non-biological scanner effects caused by different image acquisition settings. On the other hand, we study not only the Z-Score and Nyúl methods, but also the intensity normalization methods depending on WM intensities and thus dedicated to brain MRI images, including WhiteStripe, FCM-based, GMM-based and KDE-based normalization. Lastly, we also emphasize here the preciousness and rareness of our healthy volunteer dataset, in which the healthy volunteers have two MRI scans on two different scanners (Optima MR450w 1.5T and Discovery MR750w 3T) in a very short time interval (within 40 min). Unlike the clinical patient data, the differences in radiomic features can only come from the differences in image acquisition settings.

Future works may consist of several aspects. Firstly, we only investigated radiomic features extracted from the healthy white matter ROIs in clinical patient data, since we could not ensure that tumors have been developed or not between two scans. As a future work, radiomic features extracted from tumor regions should be studied to verify our conclusions regarding the image preprocessing and harmonization methods, based on a clinical radiomics problem. Secondly, in our experiments, we applied the ComBat method for each tissue class separately and proved most of the scanner effects have been removed. We also observed that if we apply ComBat for all the tissue classes together, then there were still parts of scanner effects left after applying ComBat. This can be easily explained by the inner hypothesis of the ComBat model. The ComBat method assumes each feature data follows a normal distribution if there are no covariates of interest, this hypothesis is proper when ComBat is applied for each tissue class separately, whereas each feature data sample consisting of all tissue classes usually follows a mixture of normal distributions. In addition, the additive (multiplicative) scanner effects may also be different for each feature and each tissue class, whereas if we apply the ComBat for all tissue classes together, then for each feature ComBat would assume the same additive (multiplicative) scanner effects if the feature samples come from the same scanner setting, no matter they are from the same tissue class or not. New harmonization methods should be developed to solve this problem, which may help the clinical classification problem with the class labels (such as tumor grades, the pathological types) unknown, thus making it impossible to apply the ComBat for each tumor class separately. Thirdly, the radiomic features were extracted using the fixed bin number strategy, with 32 fixed bins. The conclusions may be different for fixed bin size feature extraction strategy, or for fixed bin number strategy but with a different fixed bin number [19]. Besides, our experiments were done based on T1 sequences of the brain MRI, the results on other clinical MRI sequences may be different. So the conclusions in our paper would need to be further assessed on other independent datasets, on other clinical MRI sequences, on some radiomic models with clinical problems to be solved and so on.

## 5. Conclusions

In this paper, we studied the influences of the image preprocessing methods (N4 bias field correction and image resampling) and the harmonization methods (intensity normalization methods working on brain MRI images, and ComBat method working on radiomic features) on the reproducibility of brain MRI radiomic features using both in vitro and in vivo data, with scanner effects introduced by different magnetic field strengths, FOVs and matrices. Based on our analysis, although intensity normalization methods can make the MRI images standardized and comparable, they cannot remove the scanner effects in the radiomic feature level. ComBat method is essential and the key factor to remove scanner effects existing in radiomic features, with the non-biological information removed and the useful biological information well preserved. Besides, the intensity normalization methods make the harmonization results more robust on choosing different variants of the ComBat method, namely, standard ComBat, parametric ComBat and non-parametric ComBat.

## Data Availability

The data presented in this study are available on request from the corresponding author. The data are not publicly available due to privacy and ethical concerns.

## Abbreviations

The following abbreviations are used in this manuscript:

MRI	Magnetic Resonance Imaging
HU	Hounsfield Unit
CT	Computed Tomography
SUV	Standardized Uptake Value
PET	Positron Emission Tomography
WM	White Matter
GM	Gray Matter
CC	Corpus Callosum
WS	WhiteStripe
FCM	Fuzzy C-Means
GMM	Gaussian Mixture Model
KDE	Kernel Density Estimate
HRCT	High-Resolution Computed Tomography
FOV	Field of View
ROI	Region of Interest
EB	Empirical Bayes
SNR	Signal-to-Noise Ratio
1.5T	1.5 Tesla
3T	3 Tesla
FLAIR	Fluid-Attenuated Inversion Recovery
ICC	Intraclass Correlation Coefficient
PCA	Principal Component Analysis

## References

[1] Kumar V., Gu Y., Basu S., Berglund A., Eschrich S.A., Schabath M.B., Forster K., Aerts H.J., Dekker A., Fenstermacher D. (2012). Radiomics: The process and the challenges. Magn. Reson. Imaging.

[2] Gillies R.J., Kinahan P.E., Hricak H. (2016). Radiomics: Images are more than pictures, they are data. Radiology.

[3] Rizzo S., Botta F., Raimondi S., Origgi D., Fanciullo C., Morganti A.G., Bellomi M. (2018). Radiomics: The facts and the challenges of image analysis. Eur. Radiol. Exp..

[4] Cameron A., Khalvati F., Haider M.A., Wong A. (2015). MAPS: A quantitative radiomics approach for prostate cancer detection. IEEE Trans. Biomed. Eng..

[5] Li H., Zhu Y., Burnside E.S., Huang E., Drukker K., Hoadley K.A., Fan C., Conzen S.D., Zuley M., Net J.M. (2016). Quantitative MRI radiomics in the prediction of molecular classifications of breast cancer subtypes in the TCGA/TCIA data set. NPJ Breast Cancer.

[6] Berenguer R., Pastor-Juan M.D.R., Canales-Vázquez J., Castro-García M., Villas M.V., Mansilla Legorburo F., Sabater S. (2018). Radiomics of CT features may be nonreproducible and redundant: Influence of CT acquisition parameters. Radiology.

[7] Park J.E., Park S.Y., Kim H.J., Kim H.S. (2019). Reproducibility and generalizability in radiomics modeling: Possible strategies in radiologic and statistical perspectives. Korean J. Radiol..

[8] Fortin J.P., Cullen N., Sheline Y.I., Taylor W.D., Aselcioglu I., Cook P.A., Adams P., Cooper C., Fava M., McGrath P.J. (2018). Harmonization of cortical thickness measurements across scanners and sites. Neuroimage.

[9] Buch K., Kuno H., Qureshi M.M., Li B., Sakai O. (2018). Quantitative variations in texture analysis features dependent on MRI scanning parameters: A phantom model. J. Appl. Clin. Med. Phys..

[10] Baeßler B., Weiss K., Dos Santos D.P. (2019). Robustness and reproducibility of radiomics in magnetic resonance imaging: A phantom study. Investig. Radiol..

[11] Lee J., Steinmann A., Ding Y., Lee H., Owens C., Wang J., Yang J., Followill D., Ger R., MacKin D. (2021). Radiomics feature robustness as measured using an MRI phantom. Sci. Rep..

[12] Ammari S., Pitre-Champagnat S., Dercle L., Chouzenoux E., Moalla S., Reuze S., Talbot H., Mokoyoko T., Hadchiti J., Diffetocq S. (2021). Influence of Magnetic Field Strength on Magnetic Resonance Imaging Radiomics Features in Brain Imaging, an in vitro and in vivo Study. Front. Oncol..

[13] Lerski R., Schad L., Luypaert R., Amorison A., Muller R., Mascaro L., Ring P., Spisni A., Zhu X., Bruno A. (1999). Multicentre magnetic resonance texture analysis trial using reticulated foam test objects. Magn. Reson. Imaging.

[14] Mayerhoefer M.E., Szomolanyi P., Jirak D., Materka A., Trattnig S. (2009). Effects of MRI acquisition parameter variations and protocol heterogeneity on the results of texture analysis and pattern discrimination: An application-oriented study. Med. Phys..

[15] Jirák D., Dezortová M., Hájek M. (2004). Phantoms for texture analysis of MR images. Long-term and multi-center study. Med. Phys..

[16] Benoit-Cattin H. (2006). Texture Analysis for Magnetic Resonance Imaging.

[17] Nyúl L.G., Udupa J.K., Zhang X. (2000). New variants of a method of MRI scale standardization. IEEE Trans. Med Imaging.

[18] Shinohara R.T., Sweeney E.M., Goldsmith J., Shiee N., Mateen F.J., Calabresi P.A., Jarso S., Pham D.L., Reich D.S., Crainiceanu C.M. (2014). Statistical normalization techniques for magnetic resonance imaging. Neuroimage Clin..

[19] Carré A., Klausner G., Edjlali M., Lerousseau M., Briend-Diop J., Sun R., Ammari S., Reuzé S., Andres E.A., Estienne T. (2020). Standardization of brain MR images across machines and protocols: Bridging the gap for MRI-based radiomics. Sci. Rep..

[20] Pinto M.S., Paolella R., Billiet T., Van Dyck P., Guns P.J., Jeurissen B., Ribbens A., den Dekker A.J., Sijbers J. (2020). Harmonization of brain diffusion MRI: Concepts and methods. Front. Neurosci..

[21] Reinhold J.C., Dewey B.E., Carass A., Prince J.L. (2019). Evaluating the impact of intensity normalization on MR image synthesis. Proc. SPIE.

[22] Dewey B.E., Zhao C., Reinhold J.C., Carass A., Fitzgerald K.C., Sotirchos E.S., Saidha S., Oh J., Pham D.L., Calabresi P.A. (2019). DeepHarmony: A deep learning approach to contrast harmonization across scanner changes. Magn. Reson. Imaging.

[23] Johnson W.E., Li C., Rabinovic A. (2007). Adjusting batch effects in microarray expression data using empirical Bayes methods. Biostatistics.

[24] Andrearczyk V., Depeursinge A., Müller H. (2019). Learning cross-protocol radiomics and deep feature standardization from CT images of texture phantoms. Proc. SPIE.

[25] Chatterjee A., Vallières M., Dohan A., Levesque I.R., Ueno Y., Saif S., Reinhold C., Seuntjens J. (2019). Creating robust predictive radiomic models for data from independent institutions using normalization. IEEE Trans. Radiat. Plasma Med. Sci..

[26] Da-Ano R., Masson I., Lucia F., Doré M., Robin P., Alfieri J., Rousseau C., Mervoyer A., Reinhold C., Castelli J. (2020). Performance comparison of modified ComBat for harmonization of radiomic features for multicenter studies. Sci. Rep..

[27] Radua J., Vieta E., Shinohara R., Kochunov P., Quidé Y., Green M.J., Weickert C.S., Weickert T., Bruggemann J., Kircher T. (2020). Increased power by harmonizing structural MRI site differences with the ComBat batch adjustment method in ENIGMA. NeuroImage.

[28] Garcia-Dias R., Scarpazza C., Baecker L., Vieira S., Pinaya W.H., Corvin A., Redolfi A., Nelson B., Crespo-Facorro B., McDonald C. (2020). Neuroharmony: A new tool for harmonizing volumetric MRI data from unseen scanners. NeuroImage.

[29] Chen A.A., Beer J.C., Tustison N.J., Cook P.A., Shinohara R.T., Shou H. (2020). Removal of scanner effects in covariance improves multivariate pattern analysis in neuroimaging data. bioRxiv.

[30] Beer J.C., Tustison N.J., Cook P.A., Davatzikos C., Sheline Y.I., Shinohara R.T., Linn K.A., Initiative A.D.N. (2020). Longitudinal combat: A method for harmonizing longitudinal multi-scanner imaging data. Neuroimage.

[31] Fortin J.P., Parker D., Tunç B., Watanabe T., Elliott M.A., Ruparel K., Roalf D.R., Satterthwaite T.D., Gur R.C., Gur R.E. (2017). Harmonization of multi-site diffusion tensor imaging data. Neuroimage.

[32] Orlhac F., Boughdad S., Philippe C., Stalla-Bourdillon H., Nioche C., Champion L., Soussan M., Frouin F., Frouin V., Buvat I. (2018). A postreconstruction harmonization method for multicenter radiomic studies in PET. J. Nucl. Med..

[33] Orlhac F., Frouin F., Nioche C., Ayache N., Buvat I. (2019). Validation of a method to compensate multicenter effects affecting CT radiomics. Radiology.

[34] Mahon R., Ghita M., Hugo G., Weiss E. (2020). ComBat harmonization for radiomic features in independent phantom and lung cancer patient computed tomography datasets. Phys. Med. Biol..

[35] Saint Martin M.J., Orlhac F., Akl P., Khalid F., Nioche C., Buvat I., Malhaire C., Frouin F. (2020). A radiomics pipeline dedicated to Breast MRI: Validation on a multi-scanner phantom study. Magn. Reson. Mater. Phys. Biol. Med..

[36] Arendt C.T., Leithner D., Mayerhoefer M.E., Gibbs P., Czerny C., Arnoldner C., Burck I., Leinung M., Tanyildizi Y., Lenga L. (2020). Radiomics of high-resolution computed tomography for the differentiation between cholesteatoma and middle ear inflammation: Effects of post-reconstruction methods in a dual-center study. Eur. Radiol..

[37] Orlhac F., Lecler A., Savatovski J., Goya-Outi J., Nioche C., Charbonneau F., Ayache N., Frouin F., Duron L., Buvat I. (2020). How can we combat multicenter variability in MR radiomics? Validation of a correction procedure. Eur. Radiol..

[38] Um H., Tixier F., Bermudez D., Deasy J.O., Young R.J., Veeraraghavan H. (2019). Impact of image preprocessing on the scanner dependence of multi-parametric MRI radiomic features and covariate shift in multi-institutional glioblastoma datasets. Phys. Med. Biol..

[39] Moradmand H., Aghamiri S.M.R., Ghaderi R. (2020). Impact of image preprocessing methods on reproducibility of radiomic features in multimodal magnetic resonance imaging in glioblastoma. J. Appl. Clin. Med. Phys..

[40] Bologna M., Corino V., Mainardi L. (2019). Virtual phantom analyses for preprocessing evaluation and detection of a robust feature set for MRI-radiomics of the brain. Med. Phys..

[41] Scalco E., Belfatto A., Mastropietro A., Rancati T., Avuzzi B., Messina A., Valdagni R., Rizzo G. (2020). T2w-MRI signal normalization affects radiomics features reproducibility. Med. Phys..

[42] Shiri I., Hajianfar G., Sohrabi A., Abdollahi H.P., Shayesteh S., Geramifar P., Zaidi H., Oveisi M., Rahmim A. (2020). Repeatability of radiomic features in magnetic resonance imaging of glioblastoma: Test–retest and image registration analyses. Med. Phys..

[43] Hoebel K.V., Patel J.B., Beers A.L., Chang K., Singh P., Brown J.M., Pinho M.C., Batchelor T.T., Gerstner E.R., Rosen B.R. (2020). Radiomics Repeatability Pitfalls in a Scan-Rescan MRI Study of Glioblastoma. Radiol. Artif. Intell..

[44] Avants B.B., Tustison N.J., Stauffer M., Song G., Wu B., Gee J.C. (2014). The Insight ToolKit image registration framework. Front. Neuroinform..

[45] Klein A., Andersson J., Ardekani B.A., Ashburner J., Avants B., Chiang M.C., Christensen G.E., Collins D.L., Gee J., Hellier P. (2009). Evaluation of 14 nonlinear deformation algorithms applied to human brain MRI registration. Neuroimage.

[46] Murphy K., Van Ginneken B., Reinhardt J.M., Kabus S., Ding K., Deng X., Cao K., Du K., Christensen G.E., Garcia V. (2011). Evaluation of registration methods on thoracic CT: The EMPIRE10 challenge. IEEE Trans. Med. Imaging.

[47] Zwanenburg A., Vallières M., Abdalah M.A., Aerts H.J., Andrearczyk V., Apte A., Ashrafinia S., Bakas S., Beukinga R.J., Boellaard R. (2020). The image biomarker standardization initiative: Standardized quantitative radiomics for high-throughput image-based phenotyping. Radiology.

[48] Griethuysen J.J.M., Fedorov A., Parmar C., Hosny A., Aucoin N., Narayan V., Beets-Tan R.G.H., Fillon-Robin J.C., Pieper S., Aerts H.J.W.L. (2017). Computational radiomics system to decode the radiographic phenotype. Cancer Res..

[49] Tustison N.J., Avants B.B., Cook P.A., Zheng Y., Egan A., Yushkevich P.A., Gee J.C. (2010). N4ITK: Improved N3 bias correction. IEEE Trans. Med. Imaging.

[50] Shah M., Xiao Y., Subbanna N., Francis S., Arnold D.L., Collins D.L., Arbel T. (2011). Evaluating intensity normalization on MRIs of human brain with multiple sclerosis. Med. Image Anal..

[51] Friedman M. (1940). A comparison of alternative tests of significance for the problem of m rankings. Ann. Math. Stat..

[52] Wilcoxon F. (1992). Individual comparisons by ranking methods. Breakthroughs in Statistics.

[53] Neyman J., Pearson E.S. (2020). On the Use and Interpretation of Certain Test Criteria for Purposes of Statistical Inference.

[54] Dunn O.J. (1961). Multiple comparisons among means. J. Am. Stat. Assoc..

[55] Armstrong R.A. (2014). When to use the Bonferroni correction. Ophthalmic Physiol. Opt..

[56] Wold S., Esbensen K., Geladi P. (1987). Principal component analysis. Chemom. Intell. Lab. Syst..

[57] Abdi H., Williams L.J. (2010). Principal component analysis. Wiley Interdiscip. Rev. Comput. Stat..

